# Age-Related Immune Response to Previous Smallpox Vaccination in Albanian Subjects and Cross-Reactivity Between Vaccinia Virus and Monkeypox Virus Antigens

**DOI:** 10.3390/vaccines14100889

**Published:** 2026-10-10

**Authors:** Adriana Manuka, Irsida Mehmeti, Antonia Radaelli, Hamide Shllaku-Sefa, Ndok Marku, Eduart Hashorva, Carlo Zanotto, Carlo De Giuli Morghen

**Affiliations:** 1 Faculty of Pharmacy, Catholic University “Our Lady of Good Counsel”, 1000 Tirana, Albania; 2 Department of Medical Biotechnologies and Translational Medicine, State University of Milano, 20122 Milan, Italy; 3 Laboratory Department, Catholic Hospital “Our Lady of Good Counsel”, 1023 Tirana, Albania

**Keywords:** mpox virus, antibody, smallpox vaccination, cross-reactivity, serological ELISA

## Abstract

**Background/Objectives**: Recent outbreaks of the mpox virus (MPVX) have highlighted the threat posed by the *Orthopoxvirus* (OPXV) genus, which includes variola, the causative agent of smallpox. Since routine Vaccinia virus (VACV) vaccination campaigns terminated, the majority of the global population currently lacks immunity against OPXVs. **Methods**: Using enzyme-linked immunosorbent assays (ELISAs), this study evaluated antibody titers against both VACV proteins and their MPVX orthologs across various age groups. Cross-reactivity was analyzed using 220 serum samples collected from Albanian individuals between November 2023 and January 2024, arranged into five age cohorts. We measured plasma antibody levels against whole VACV, as well as specific surface antigens: L1, B5, A33, and A27 for VACV, and M1, H3, A35, and A29 for MPXV. **Results**: The data reveal a long-term, gender-independent immunological memory targeting both whole-virus particles and individual viral proteins. **Conclusions**: Notably, the oldest cohort exhibited higher antibody titers against specific single viral proteins, whereas the younger vaccinated groups showed stronger activity against the whole virus. Additionally, antibodies raised via VACV vaccination recognize MPXV ortholog proteins equally well. This cross-reactivity highlights the broad protective potential of VACV immunization across the entire *Orthopoxvirus* genus.

## 1. Introduction

Mpox (formerly Monkeypox), a smallpox-like zoonotic disease endemic to Central and Western Africa, is caused by the monkeypox virus (MPXV), a double-stranded DNA virus of the Poxviridae family [1]. Like Variola virus (VARV), MPXV belongs to the *Orthopoxvirus* (*OPXV*) genus [2] and replicates exclusively in the cytoplasm.

After the first human case, identified in 1970 in the Democratic Republic of Congo (DRC), several mpox outbreaks have been reported in Africa. In the last two decades, sporadic outbreaks in non-endemic countries with increased human-to-human transmission have been observed.

Phylogenetically, MPXV is divided into two major clades: Clade I, formerly known as Central African, also known as the Congo Basin clade, which includes Subclades Ia and Ib; and Clade II, formerly known as West African, which includes Subclades IIa and IIb [3]. Clade I is generally associated with higher pathogenicity and mortality, with a case fatality rate of up to 10%, compared with 1% for Clade II [4]. Subclade Ib emerged in the Eastern Democratic Republic of Congo (DRC) and was associated with large-scale mpox outbreaks in 2024. Subclade IIb, which has circulated among humans since 2016, was associated with the global mpox outbreak in 2022 [5]. In contrast, Subclades Ia and IIa have been less frequently associated with sustained human transmission.

The outbreak of novel clusters of MPXV in endemic and non-endemic countries in 2022–2024 led the World Health Organization (WHO) to declare mpox a public health emergency of international concern (PHEIC) [6,7], until the emergency was lifted in September 2025, due to declining global case numbers [8]. Vaccination of high-risk groups is considered an important control measure to prevent mpox and reduce the severity of disease symptoms [8].

Currently, licensed vaccines for prophylactic protection against mpox infection are based on live-attenuated or modified Vaccinia virus (VACV) such as ACAM2000, MVA-BN, and LC16 [9].

Surveillance data from the 1980s suggest that humans vaccinated with first-generation VACV vaccines possess around 85% protection also against mpox [10]. VACV and MPXV share over 95% sequence homology, depending on strain, with a highly conserved gene and protein sequence, which may suggest immune cross-reactivity [11,12]. Notably, Ahmed et al. found around 84% genetic similarity between VACV and the 2022-strain MPXV [13]. However, following the global smallpox declaration of eradication in 1980, routine vaccination against smallpox was discontinued. As a result, herd immunity to MPXV and other OPXVs waned over the decades, leading to the increase in the number of OPXV-naïve individuals worldwide [10,14].

Insight into the humoral immune response elicited by VACV vaccination is essential to evaluate the persistence of specific antibody responses in populations vaccinated over 45 years and identify potential antigens as candidates for new vaccines.

MPXV has two antigenically distinct infectious forms: the mature virion (MV) and the enveloped virion (EV) [15]. MVs have a single membrane and assemble in the cytoplasm of virus-infected cells, whereas EVs have an additional outer membrane that facilitates both direct transfer between cells and remote virus transmission within hosts [16]. Notably, six MPXV surface proteins (H3, E8, M1, and A29 MV, and A35 and B6 EV proteins) were previously reported to elicit neutralizing antibodies and are therefore considered potential vaccine antigens [17]. These proteins are orthologs of VACV H3, D8, and L1 MV proteins and A33 and B5 EV proteins [18]. Some studies also indicate that antibodies raised against a single protein offer less protection than a combination of multiple proteins, since the surface antigen proteins of MVs and EVs are highly antigenically distinct [18,19]. Several studies have reported that B5, A33, L1, and A27 proteins are effective candidates for the development of poxvirus subunit [20,21,22] and recombinant vaccines [12,23]. These antigens have efficiently induced cross-neutralizing and protective human antibodies against poxvirus infections [20,21,22]. Recombinant vaccines expressing these proteins have also been successfully combined with DNA vaccines to enhance the immune response in putative vaccines against OPVXs [24,25,26,27,28].

The *E8L*, *H3L*, *A29L*, *A35R*, *B6R*, and *M1R* MPXV genes were also used for the development of mRNA vaccine candidates against mpox [29,30,31,32,33,34,35]. Two of these mRNA vaccines are in early-phase clinical trials: BioNTech’s BNT166a (NCT05988203), which encodes four MPXV antigens (A35, B6, M1, and H3), and Moderna’s mRNA-1769 (NCT05995275), which encodes four MPXV antigens (A35, B6, M1, and A29) [36] (Table 1).

In vivo studies showed that both anti-EV and anti-MV antibodies may be important for protection [43,44,45]. Thus, it is important to measure human antibody responses to EV, as well as to MV, when analyzing antibody titers elicited by VACV vaccination.

This study aims to evaluate the antibody response in the sera of Albanian subjects aged 44 to 93, previously vaccinated against smallpox, to determine if they still retained specific humoral immunity. The humoral response was assessed both against the whole VACV and against the single EV and MV proteins to determine the cross-reactivity of antibodies against VACV and MPXV antigens. The different humoral response in females vs. males was also evaluated. Non-immunized subjects aged 20 to 43 were also used as negative controls.

## 2. Materials and Methods

### 2.1. Study Setting

Albanian participants were recruited during routine examinations at “Our Lady of Good Counsel” Hospital and “Luigi Monti” Ambulance Service. The experiments were performed in collaboration between the NATO Laboratory of Cellular and Molecular Virology in Tirana and the Laboratory of Molecular Virology and Recombinant Vaccines Development at the University of Milan.

### 2.2. Study Participants

A total of 220 Albanian participants of both genders, aged over 20 years and up to 93 years, were included in the study. Participants aged 20 to 43 were used as negative controls. Vaccination during this period of time was mandatory, and Albanian people recruited by two clinical centers at the Hospital of Tirana could not decline this duty. Most participants also declared that they had a scar after vaccination. The sample size was calculated using the standard formula for estimating a population proportion with finite population correction (FPC). The infinite baseline (*n*_0_) was calculated using a 95% confidence level (*Z* = 1.96), an expected proportion of 50% (*p* = 0.50), and a margin of error of 5% (e = 0.05) with Cochran’s formula:

$$n_{0}=\frac{Z^{2}p\left(1-p\right)}{e^{2}}$$

The value of 50% was selected as a conservative estimate, because no reliable prior estimate of the expected proportion was available. The finite population correction (FPC) was then calculated and applied using the formula (*n* = *n*_0_/[1 + (*n*_0_ − 1)/N]), where N represents the total population size. The target population size of approximately 400 individuals (N ≈ 400) was defined as the total number of eligible individuals who met the study inclusion criteria and were enrolled at the two clinical centers during the study recruitment period. Based on the initial unadjusted sample size of n_0_ = 384, assuming a 50% response rate, a 95% confidence level, and a 5% margin of error, the finite population correction (FPC) formula [*n* = *n*_0_/(1 + (*n*_0_ − 1)/N)] resulted in a minimum required sample size of *n* = 196 participants.

### 2.3. Experimental Design

The participants were divided into four age groups, each group consisting of 50 participants. Group A included 44–53-year-old individuals, born between 1970 and 1979; Group B included 54–63-year-old individuals, born between 1960 and 1969; Group C included 64–73-year-old individuals, born between 1950 and 1959; and Group D included over 74-year-old individuals, born between 1930 and 1949 (Table 2).

Among the participants, 61% were women and 39% were men. An additional group of 20 individuals including 20–43-year-old individuals, born after the smallpox vaccination era, i.e., from 1980 to 2004, were used as negative controls (Group 0). Subjects with a history of recent or chronic infectious diseases, especially with sexually transmitted diseases of viral origin, were not considered eligible for this study. Subjects with known immunosuppressive conditions were also excluded.

The antibody response was determined according to the titers elicited by the different participants against the inactivated VACV, or against either the four single L1, B5, A27 and A33 VACV proteins or the four single M1, H3, A29 and A35 MPXV proteins.

### 2.4. Blood Samples

Blood samples were collected by venipuncture using VacuMed gel and clot activator tubes (FL Medical, Padua, Italy). After 2 h at room temperature, samples were spun on an ECOspin medical centrifuge (ECONet Gmbh, Oberhausen, Germany) for 10 min at 4000 rpm. Aliquots of the sera were stored at −20 °C until analysis. Serum samples showing visible hemolysis were excluded from the analysis.

### 2.5. Cells

Vero cells, derived from green monkey kidney, were grown in Dulbecco’s modified Eagle’s medium (DMEM, Gibco, Life Technologies, Rockville, MD, USA) supplemented with 10% heat-inactivated calf serum (Newborn Calf Serum, CS, Gibco/Invitrogen, Carlsbad, CA, USA), 100 U/mL penicillin, and 100 mg/mL streptomycin (P/S).

### 2.6. Virus Preparation and Inactivation

The Lederle strain of VACV, employed for human vaccination and distributed by Sclavo Company, was obtained through ISM (Istituto Sieroterapico Milanese). Infection and amplification of the virus were conducted on Vero cells within a high-containment facility. Purification was carried out using high-speed sucrose gradient centrifugation [24]. Virus inactivation was performed utilizing UV and psoralen as already described, with minor modifications [46,47,48]. Briefly, 10 μg/mL psoralen in Hanks balanced salt solution was added to the virus (8 × 10^8^ PFU/mL) and incubated in a 6-well dish for 10 min at room temperature (1 mL/well) under UV lamps (366 nm) at a distance of 2 cm for 12 min (4 exposures of 3 min each, with a 1 min interval). Viral titration was performed both prior to and following inactivation to verify the absence of infectious virus.

### 2.7. Enzyme-Linked Immunosorbent Assay

Serum samples were analyzed for antibody response to VACV using enzyme-linked immunosorbent assays (ELISAs). The assay employed plate-bound antigens, which included purified inactivated whole VACV, individual VACV proteins, and individual MPXV proteins.

Specifically, the inactivated VACV (2 × 10^8^ PFU/mL) was plated 100 μL/well onto 96-well microtiter plates (MaxiSorp; Nunc, ThermoScientific, Roskilde, Denmark) in 0.05 M carbonate–bicarbonate buffer, pH 9.6. A mixture of the L1, B5, A33, and A27 VACV proteins as well as a mixture of the M1, H3, A35 and A29 MPXV proteins, kindly provided by the Biodefense and Emerging Infections Research Resources Repository (BEI Resources, NIAID, NIH, Manassas, VA, USA), were separately coated onto MaxiSorp plates in triplicate, 100 ng/well or 40 ng/well, respectively, in 100 µL Ca^2+^- and Mg^2+^-free Phosphate-Buffered Saline (PBS), pH 7.2 [49]. All plates were incubated overnight at 4 °C. Diluted sera from vaccinated individuals or from Group 0 were incubated for 1 h at 37 °C.

Detection was performed using a goat anti-human horseradish peroxidase-conjugated secondary antibody at 1:1000 or 1:2000 dilutions (Invitrogen ThermoScientific, Rockford, IL, USA) for 1.5 h. The enzymatic reaction was developed by adding 3,3′,5,5′-tetramethyl-benzidine substrate (TMB, ThermoScientific, Rockford, IL, USA) and blocked with 2 M H_2_SO_4_ for 30 min. Absorbance at 450 nm was immediately measured using a microplate reader (Stat Fax 4200; Awareness Technology, Inc., Palm City, FL, USA). Along with the sera from unvaccinated subjects of Group 0, a panel of sera from fifteen known negative controls was tested in triplicate. This panel included two sera from unvaccinated Albanian children, aged 2 and 4 years, with no history of OPXV exposure, five human sera provided by the Laboratory of Molecular Virology and Recombinant Vaccine Development (University of Milan), and eight human sera kindly provided by Prof. A. Manenti (Chief Development Officer, VisMederi Srl, Siena, Italy).

For all ELISAs, both when humoral immunity was determined against the whole virus or against the single proteins, the O.D. of the negative controls was 0.060 and the assay cut-off was set as twice this mean, i.e., 0.120 O.D. Thus, samples with O.D. equal to or above this threshold were considered positive, whereas those with values below 0.120 O.D. were considered negative.

### 2.8. Statistical Analysis

All data were analyzed using IBM SPSS Statistics, Version 22.0 (IBM Corp., Armonk, NY, USA). Group comparisons were conducted with the chi-square test (χ^2^), or Fisher’s exact test when suitable. Z-test was used to compare the proportions of females and males. If the comparisons did not reveal any significance, we specified it in the legend. Bonferroni correction was applied to adjust the significance threshold for multiple comparisons, with the adjusted α level computed as 0.05 divided by the total number of comparisons. A total of 24 pairwise comparisons (k = 24) were conducted among the four age groups for each of the four VACV and MPXV proteins tested. The Bonferroni-adjusted alpha level was calculated as 0.05 divided by 24, resulting in 0.0021.

Statistical significance was set using Bonferroni-adjusted *p*-values and presented as *p adj* < 0.05 (*), *p adj* < 0.01 (**), and *p adj* < 0.001 (***).

### 2.9. Ethical Considerations

The procedures performed in this study were conducted in accordance with the ethical standards of the Catholic University “Our Lady of Good Counsel”, Tirana, Albania (Prot. No. 361, 27 July 2023), the World Medical Association’s Declaration of Helsinki, ICH/GCP guidelines, and International Organization for Standardization (ISO, no. 14155, Clinical investigation of medical devices for human subjects—Good clinical practice Geneva, Switzerland, 2011.

Before sample collection, participants were informed about the aims and goals of the study and were required to provide both verbal and written informed consent. Confidentiality and anonymity of the data were ensured by assigning a progressive number to each subject.

## 3. Results

### 3.1. The Antibody Response Was Significantly Greater Against the Whole VACV in More Recently Vaccinated Individuals

When the antibody titer was examined against the purified inactivated whole VACV in the different age groups, most individuals showed titers of 1:4000/1:8000 (Figure 1). Significantly higher numbers at 1:8000 were found in Group A compared to Group C (*p* < 0.001).

### 3.2. A Significant Differential Distribution of Antibody Titers Was Found Across Age Groups, with Higher Titers Against B5 and A33 VACV Proteins in Groups C and D

The antibody titers against the single VACV proteins were evaluated in all groups. A higher percentage of individuals showed a 1:1600 antibody titer against the L1 VACV protein in all groups, but with significantly higher numbers found in Group D vs. A at 1:3200 (Figure 2A; *p* < 0.01). The majority of individuals had a 1:12,800 titer against the B5 protein (Figure 2B), but in Group A the most frequent titer was 1:6400, whereas in Groups C and D, 1:25,600 was significantly higher compared to Groups A and B (*p* < 0.01).

The majority of individuals in all groups showed a 1:12,800 antibody titer against the A33 protein (Figure 2C), with a significant percentage of Group D showing a 1:25,600 and 1:51,200 higher titer compared to the other three groups (Group D vs. Groups A, B, and C: *p* < 0.001 at 1:25,600 and *p* < 0.01 at 1:51,200). The 1:3200–1:12,800 titers were the most frequently observed against the A27 protein across all of the groups (Figure 2D), where Group A comprised an increase in the percentage of individuals showing a 1:3200 titer compared to the other groups.

### 3.3. Antibody Titers Were Higher Against A35 MPXV Protein Across Groups, with Different Responses to M1, H3, and A29 in Groups B and D

Antibody titers were also evaluated in all of the age groups against the different single MPXV antigenic proteins. Most individuals showed antibody titers against the M1 protein at 1:3200, with a higher percentage in Group D at 1:3200 vs. the other groups and in Groups B and D at 1:6400 and 12,800 (Figure 3A). The 1:3200 titer was also the most frequently observed against the H3 protein across all of the groups (Figure 3B), with a slightly higher number of subjects in Group D. Groups B and D also showed a higher percentage of individuals showing 1:6400 and 1:12,800 titers compared to Groups A and C. Regarding A35, the most common titer in Groups A, B, and C was 1:6400. Notably, among older individuals, the most common titer was higher. For A29, the majority of subjects in all groups reached antibody titers at 1:3200 (Figure 3D). Individuals in Groups B and D reached higher numbers at 1:6400 compared to Groups A and C.

### 3.4. Similar Antibody Titers but a Different Distribution in the Age Groups Were Found for A33/A27 VACV and A35/A29 MPXV Proteins

A comparison between the antibody titers against the A33 and A27 VACV and the A35 and A29 MPXV ortholog proteins showed that in the majority of subjects across groups, the response to A33 and A27 VACV proteins compared to the corresponding A35 and A29 MPXV orthologs was similar (Figure 4A). Notably, for A33, there were significantly higher percentages of individuals in Group D with titers at 1:25,600 and at 1:51,200 (Group D vs. A, B, and C, *p* < 0.01). Higher numbers of subjects in Group D were observed at 1:25,600 against the A35 protein. The antibody titers for A27/A29 ortholog proteins were also similar (Figure 4B), with generally higher numbers in Groups B and D vs. A and C. Titers were lower than against the A33/A35 proteins. A significantly higher number of individuals had a 1:6400 titer against the A29 protein in Groups B and D compared to Groups A and C (*p* < 0.05).

### 3.5. No Gender Bias in Antibody Titers for VACV and MPXV

The antibody titers against the L1, B5, A33, and A27 VACV proteins and against the M1, A35, A29, and H3 MPXV proteins were also analyzed for gender bias. The antibody responses were lower against the L1/M1 proteins, but they were generally similar in women and men against all of the VACV proteins across individuals (Figure 5A,B). Equally, no significant differences were observed when comparing the response of the two genders against the MPXV ortholog proteins.

## 4. Discussion

Vaccination against smallpox uses a live virus that induced a localized infection and caused a skin reaction [50,51]. Recent studies have found a persistence of antibodies towards VACV following the first-generation regimen [52,53]. Given the highly conserved amino acid sequence of OPXVs, smallpox vaccines may confer cross-protection against other OPXVs [12,23]. This is further supported by the cross-reactivity of human antibodies from individuals vaccinated or infected with VACV, CPXV, MPXV, and VARV [18]. Notably, viral envelope proteins, including L1, A27, A33, and B5, were reported to elicit neutralizing antibodies and are therefore considered promising vaccine antigens [18,23].

Records of smallpox vaccination in the Albanian population date back to the late 19th century and, in 1947, smallpox vaccination became mandatory nationwide [54]. As the country’s geopolitical and political circumstances changed, Albania used several first-generation smallpox vaccines, including those imported from Italy and the Soviet Union. In early 1954, smallpox vaccine production started in calves, and domestic production continued until 1987, when routine smallpox vaccination was officially discontinued [54]. Following the 1972 smallpox outbreak in the former Yugoslavia, particularly in Kosovo and Serbia [55], Albania launched a mandatory mass vaccination campaign targeting individuals aged 6 months to 60 years [54]. However, as domestic vaccine supplies were insufficient, 1.2 million doses of smallpox vaccine were imported from China and 175,000 doses from Italy for specific professional groups, including military and healthcare personnel [54].

After cessation of the mandatory smallpox vaccination, individuals born after 1980 were susceptible to other OPXV infections, and in recent years, mpox has gained significant attention worldwide.

Although Albania is classified as a low-risk country for mpox by the WHO [56], its location in the Western Balkans may facilitate the introduction of infectious diseases of African origin [57], including mpox, through migration, highlighting the need for strengthened surveillance and preventive measures.

In this study, the ELISA test was employed, using the inactivated whole VACV or the single proteins of VACV or MPXV as plate-bound antigens to determine and compare antibody titers in different age groups of subjects previously vaccinated against smallpox. The aim was to verify whether the antibody response to VACV vaccination resulted in VACV and MPXV cross-reactivity and long-lasting antibody titers against not only the L1, B5, A33, and A27 VACV antigens, but also the M1, A29, H3, and A35 MPXV ortholog surface proteins. Our results showed the following: (i) Group A, which included the youngest individuals from 44 to 53 years, showed a greater response against the whole VACV; (ii) the antibody titers against the B5 and A33 EV VACV proteins were higher than against the L1 and A27 MV proteins in most individuals; (iii) a similar response was found against the ortholog A33/A35 and A27/A29 protein pairs, with higher proportions in elderly people; (iv) the antibody response was similar in females and males both against VACV and MPXV proteins; (v) none of the groups became seronegative for any antigen.

When the whole inactivated VACV was used as the coating antigen, humoral immunity against smallpox showed higher numbers in the youngest people of Group A (44–53 years) compared with the other cohorts. When considering the different VACV proteins, the antibody titer against the EV proteins (A33 and B5) was significantly higher than against the MV proteins (L1 and A27) and higher numbers were found in the elderly population of Group D against L1, A33, and B5. Although we cannot explain these results, some studies have shown that smallpox vaccine-induced immunity wanes with time [58,59,60,61], whereas other studies [62,63] have highlighted that OPXV-specific antibodies can persist for decades and may remain robust even among aged individuals vaccinated in the pre-eradication era. Our data also show that reactivity to some of VACV-specific antigens increased with age.

These findings were further supported by Fenner et al. [64], who reported that senior subjects might have been exposed to natural or other poxvirus infections, or might have been vaccinated with highly immunogenic smallpox vaccine strains, such as the Russian Tashkent or Chinese TianTan strains. Only by 1971 were all public Health Institutes worldwide beginning to use vaccines containing less pathogenic strains [64].

The response to the MPXV proteins generally showed higher antibody titers against the A35 EV protein compared to M1, H3, and A29 MV proteins in at least 20% of individuals in all of the age groups. Notably, 38% of the individuals were aged 74 or more, as shown by Manenti et al. [65]. Our findings also showed higher antibody titers in Group B (aged 54 to 63) and in Group D (aged over 74) against the M1, H3, and A29 MPXV proteins, which belong to the MV form, although the reason remains unclear. This may reflect different antigenic stimulations during previous vaccination campaigns, such as vaccine types or vaccination schedules, which were implemented in earlier decades, or selective memory maintenance for MV antigens.

When comparing the specific response to the VACV/MPXV EV (A33/A35) or MV (A27/A29) antigens, similar antibody responses were observed against the ortholog antigen pairs. This may be due to the amino acid sequence similarity between VACV and MPXV, which share 93.64–98.8% homology in their surface proteins. These findings confirm that VACV vaccines may elicit a cross-reactive immune response and long-lasting cell memory against MPXV, as supported by Zhou et al. [63]. For both VACV and MPXV proteins, the antibody titer levels were higher against EV than MV antigens, and higher in elderly people. Higher antibody levels were also observed for the ortholog pairs of EV proteins A33/A35, whereas the MV ortholog pair A27/29 seemed to be less immunogenic. As mentioned, this is in agreement with previous studies indicating that surface proteins associated with EV forms are immunodominant [43,44,66].

Humoral immunity is known to be influenced by a variation in X/Y chromosome gene expression, sex hormones, immune cells, and the microbiome [67]. Previous studies have suggested that females have a stronger humoral immune response to smallpox vaccination [68,69] and may also exhibit higher T-cell immune responses against both VACV and MPXV than males [62]. However, in agreement with other studies [17,31], our results showed that the response to VACV/MPXV ortholog proteins was similar in both genders.

In this study, lower antibody levels against the VACV L1 protein were found, in agreement with the findings of Pütz et al. [70]. Although L1 is known to be immunogenic in animal models, anti-L1 titers appear to be more robust after vaccination with L1 by itself than administered in combination with other antigens [70]. Despite its ability to neutralize MV particles, its role in the human response to OPXV infections remains unclear [12]. A possible explanation for the weak anti-L1 antibody responses after smallpox vaccination may be due to the limited quantity of L1 on the MV membrane, compared with A27 and H3, which are the most abundant proteins in MV forms, as suggested by Chung et al. [71].

In accordance with other studies [72,73,74,75], our research showed that both anti-EV and anti-MV specific antibodies persist after smallpox vaccination. Dee et al. [76] also described higher antibody titers against VACV A33 and MPXV A35 antigens in individuals aged over 74 years, and a higher antibody titer was also shown against MPXV by Dos Santos et al. [77] in elderly volunteers compared with adults, young people, and children.

Therefore, our data provide further real-life evidence, indicating that the level of the humoral response against the different antigens may differ among age groups in a given population. The younger people in Group A were less responsive than those in Group D, and often Group B, where higher titers against both VACV and MPXV proteins were detected.

The greater antibody levels in older individuals compared to younger individuals might be affected by the method of administration of smallpox vaccines or by the tuberculosis status of each participant and concurrent immunizations. Vaccination of older individuals was performed through scarification rather than by injection [59,61,78], thereby engaging epidermal and dermal cells and potentially providing a more favorable cellular environment for viral replication. The scarification procedure has been employed for smallpox vaccination among individuals over 70 years. This hypothesis is further supported by historical records from the Albania State Archive [79] that describe certain mistakes. These mistakes resulted in larger scars and may have contributed to stronger immune responses and longer-lasting immunological memory. Mariotti et al. reported that individuals aged over 45 years with latent tuberculosis infection (LTBI) exhibited stronger immune memory against VACV [80], which may correlate with the high prevalence of tuberculosis in Albania before World War II [81].

Overall, our results indicate a significantly higher response of Group A to the antigens when whole VACV is used as a plate-bound antigen. However, Group D showed higher responses towards subunit antigens, which are also produced as folded proteins, although at varying degrees of fidelity, either from Baculovirus, E. coli or HEK systems. This difference might be ascribed to different recognition of linear or conformational epitopes. EV antigens elicited higher antibody responses than MV antigens which were similar against VACV and MPXV proteins.

Further studies will clarify whether the different vaccines and vaccination regimens may have stimulated different antibody responses. It is also important to determine whether higher levels of antibodies also correspond to higher levels of neutralizing antibodies. Our study confirms that vaccination with VACV can provide a humoral response to MPXV, with the potential of providing protection against infection.

### Limitations and Future Prospects

Studies in humans have shown that smallpox vaccination using VACV could generate anti-MPXV neutralizing antibodies [20,21,22]. In the present study, the neutralization assay could not be performed for the restricted laboratory use of human pathogenic viruses, so we could not establish the antibody’s functional activity and protection against mpox infection. Since MPXV and VACV share an approximately 95% sequence similarity for immune-dominant antigens, such as A35, B6, A29, and M1, it is very likely that the VACV-neutralizing monoclonal antibodies could also neutralize MPXV [31]. These antigens can be considered effective candidates against OPXVs for the development of poxvirus subunit or recombinant vaccines [12,20,21,22,23,24,25,26,27,28].

Another limitation could be the use of twice the mean O.D. of the negative controls as the cut-off in ELISA. Although this is not the only approach for cut-off determination, it is the most commonly used method for its assessment and the most reliable one.

Although second- and third-generation smallpox vaccines may play a significant role in protecting against MPXV, overcoming the lower immunogenicity, limited vaccine supply, and unknown cross-protection against some virulent strains remains instrumental to spur a wave of new strategies [82]. New vaccine platforms employ advanced technologies, including recombinant DNA, protein subunits, and mRNA vaccines [83], that deliver specific antigens and elicit humoral/cellular immune responses similar to those of live-attenuated vaccines [84]. mRNA vaccines are regarded as a promising strategy for mpox prevention [85], with two candidates in Phase I/II clinical trials [36]. However, these innovative vaccines also have some limitations, including lower immunogenicity and stability, as well as the need for an advanced delivery system, and the intrinsic inflammatory nature of mRNA represents an obstacle [86,87]. They may also reflect limitations in the manufacturing process, logistics, delivery and distribution, particularly in high-risk countries where mpox is endemic, temperature conditions are very challenging, and infrastructures are poor. It will be important to develop mpox vaccines that guarantee efficacy, safety and accessibility also in low-income countries.

Future studies should investigate whether modern vaccines can be optimized to elicit robust responses in younger populations who lack immunity from prior smallpox vaccination.

## Figures and Tables

**Figure 1:**
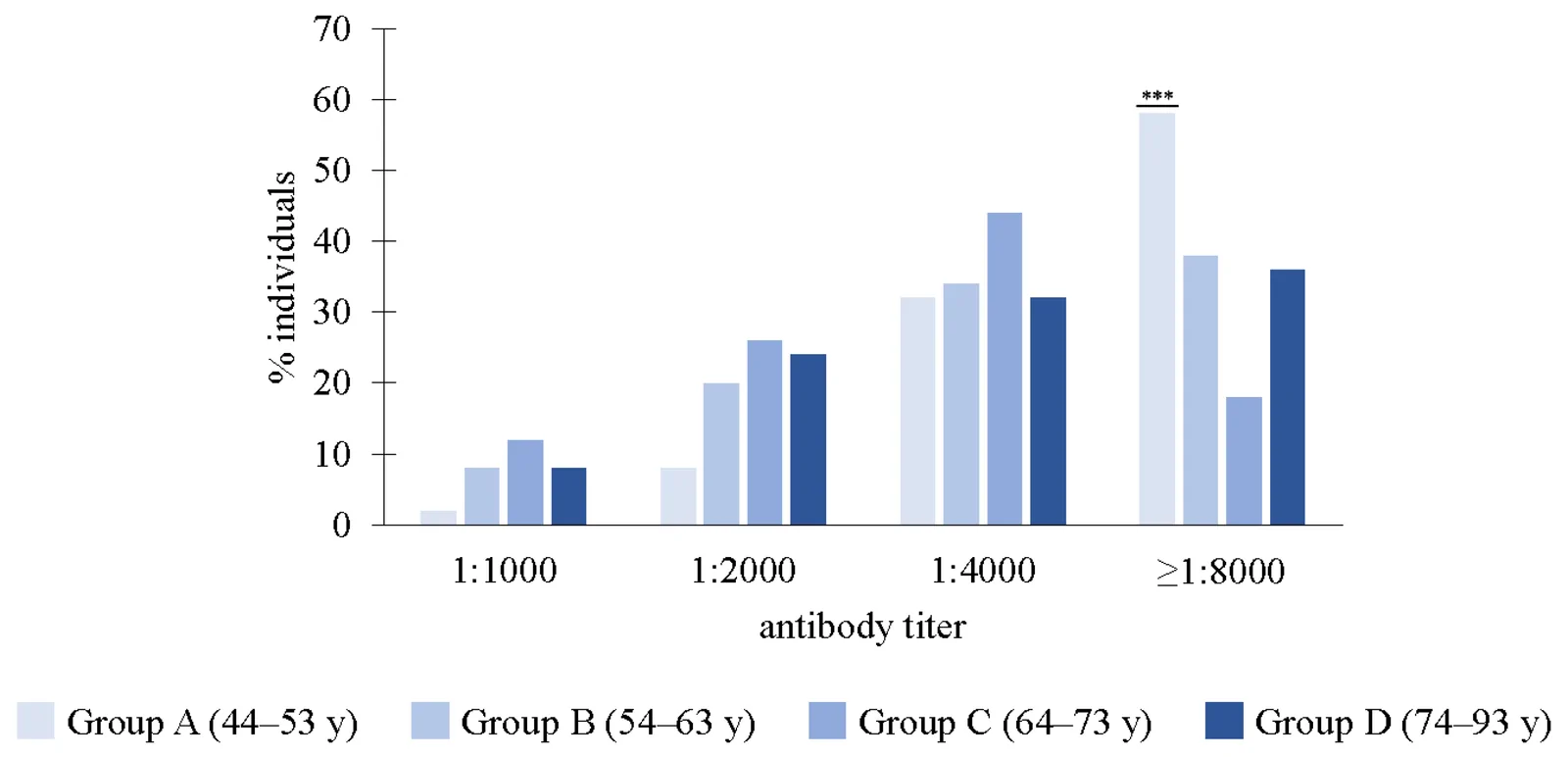
The antibody response against inactivated VACV in the different age groups. In the different age groups, most individuals showed titers at 1:4000/1:8000 against the inactivated VACV. The percentage of individuals in Group A was significantly higher compared to Group C at 1:8000 (*p* < 0.001). Statistical differences (chi-square test with Bonferroni correction) are shown as *** *p* < 0.001.

**Figure 2:**
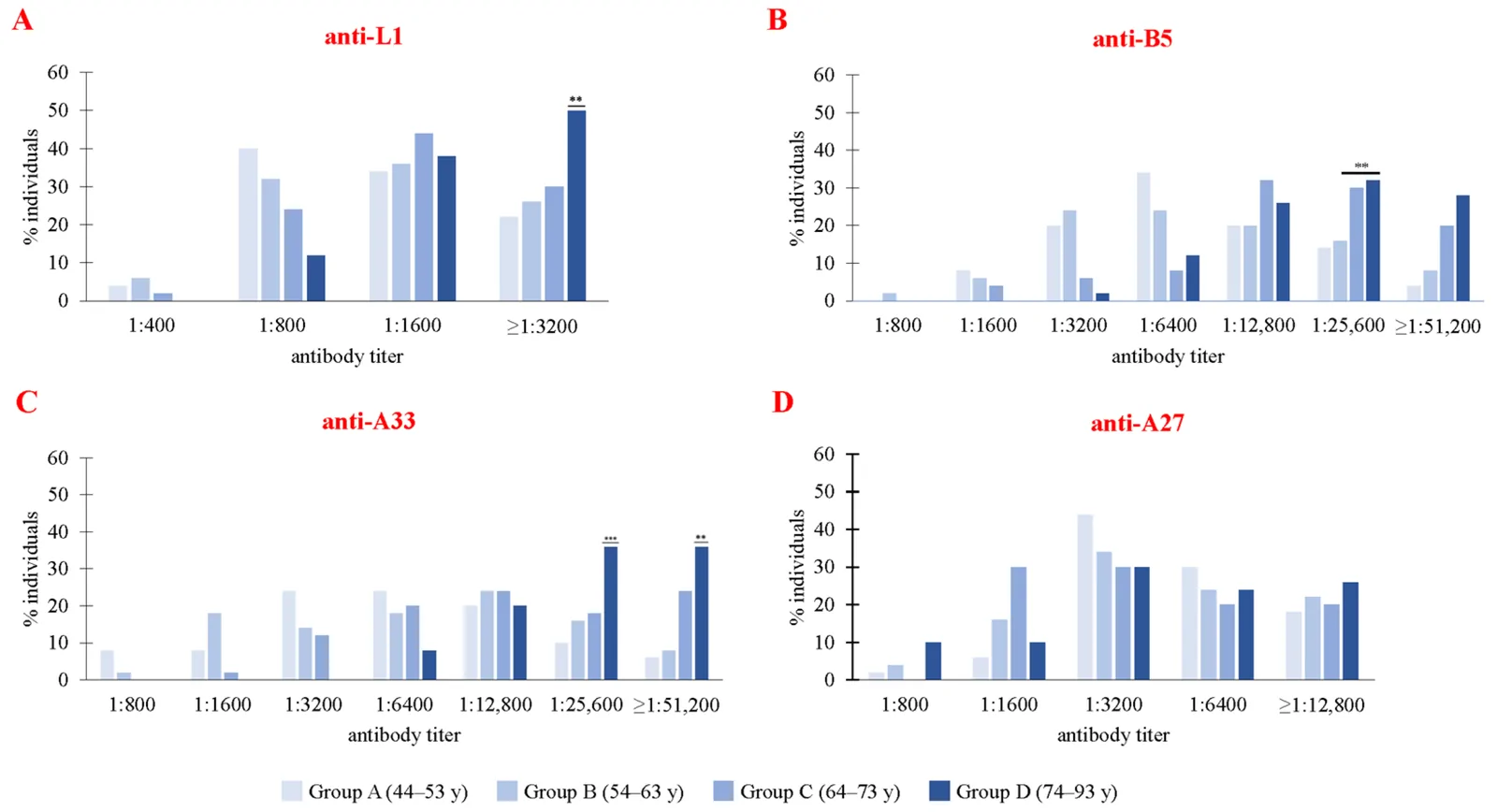
The antibody responses against VACV single proteins in the different age groups. The antibody titers against the L1, B5, A33, and A27 VACV proteins were examined. (**A**) The response against L1 was higher in most individuals at 1:1600 against all of the proteins. Significantly higher numbers were present in Group D vs. A at 1:3200 (Group D vs. A, *p* < 0.01). (**B**) Most individuals showed a 1:12,800 titer against the B5 protein, with the most frequent titers in Groups A and B at 1:3200–1:6400, whereas significantly predominant titers were found in most individuals of Groups C and D at 1:25,600 vs. Groups A and B (Groups C and D vs. A and B, *p* < 0.01). (**C**) Most individuals reached a 1:12,800 antibody titer against the A33 protein, with Group D showing a significantly higher titer at 1:25,600 and 1:51,200 vs. the other three groups (Group D vs. Groups A, B, and C: *p* < 0.001 at 1:25,600 and *p* < 0.01 at 1:51,200). (**D**) The most frequent titers against the A27 protein were 1:3200–1:12,800 across all of the groups. Group A showed a relevant increase in the percentage of individuals showing a 1:3200 titer compared to. the other groups. Statistical differences (chi-square test with Bonferroni correction) are shown: ** *p* < 0.01; *** *p* < 0.001.

**Figure 3:**
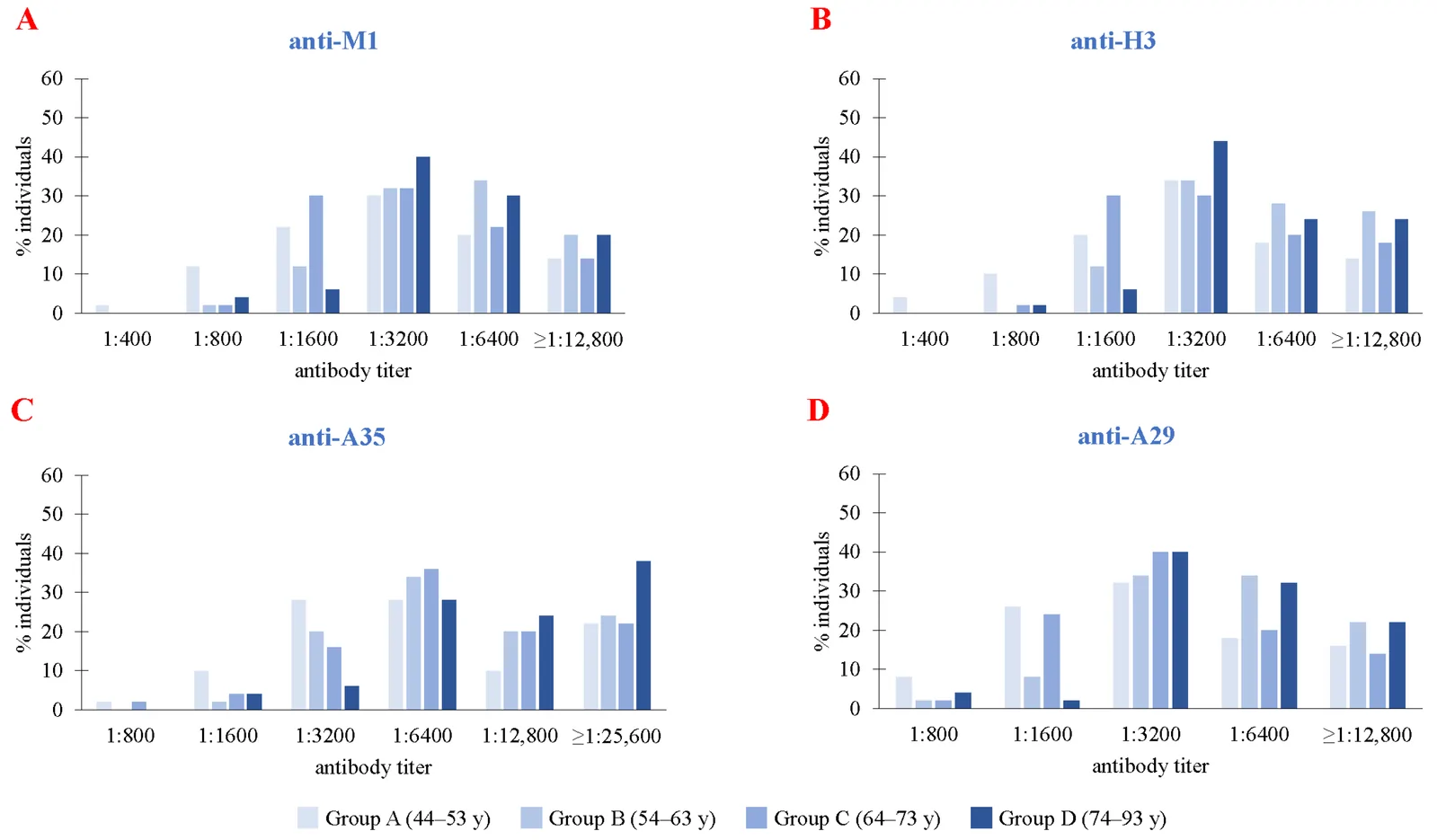
The antibody responses against MPXV single proteins in the different age groups. The antibody titers against the M1, H3, A35, and A29 MPXV proteins were examined. (**A**,**B**,**D**) The majority of individuals showed a 1:3200 antibody titer against M1, H3, and A29, with slightly higher numbers in Group D. Higher numbers were also found in Groups B and D at 1:6400 and 1:12,800 antibody titers compared to Groups A and C. (**C**) Also, higher titer levels were present in most individuals against the A35 protein compared to the other proteins, where the numbers were higher in Group D at 1:25,600. After Bonferroni correction, statistical differences among the groups were not found.

**Figure 4:**
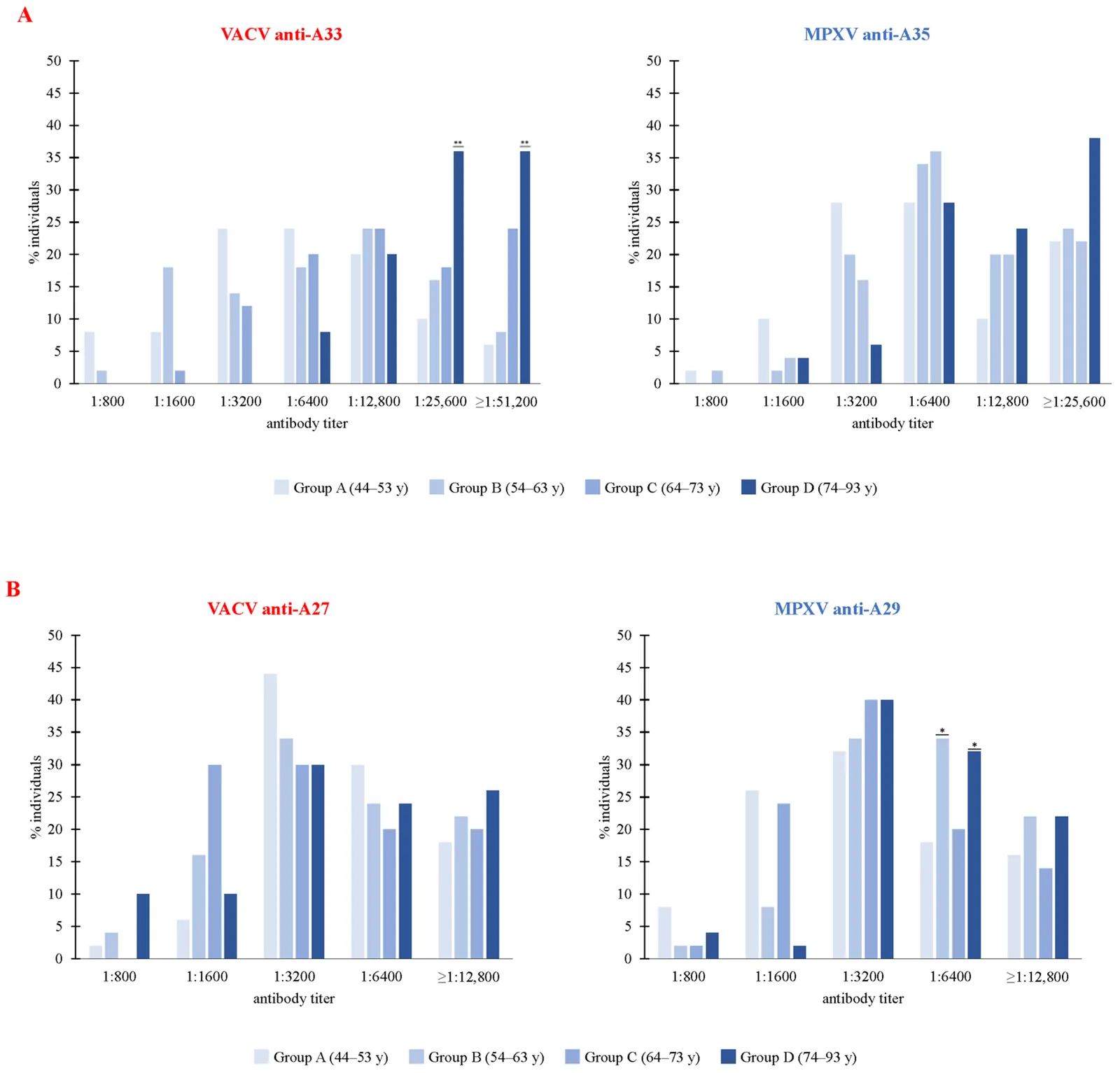
Comparison between the antibody response to VACV and MPXV proteins in the different age groups. The response to A33/A35 and A27/A29 ortholog proteins was compared. The antibody titers against the VACV A33 vs. MPXV A35 and VACV A27 vs. MPXV A29 ortholog proteins were similar in the majority of subjects in all groups. (**A**) In Group D, the numbers against the A33 VACV protein were significantly higher vs. the other groups at 1:25,600 and 1:51,200 (Group D vs. A, B, and C, *p* < 0.01). For A35, Group D reached higher numbers at 1:25,600. Lower levels were generally attained by Group A compared to the other age groups. (**B**) The response to the A27/A29 ortholog proteins was also similar, with significantly higher numbers generally reached by Groups B and D at 1:6400 against the A29 protein vs. the other groups (Groups B and D vs. A and C, *p* < 0.05). Statistical differences (chi-square test with Bonferroni correction) are shown: * *p* < 0.05; ** *p* < 0.01.

**Figure 5:**
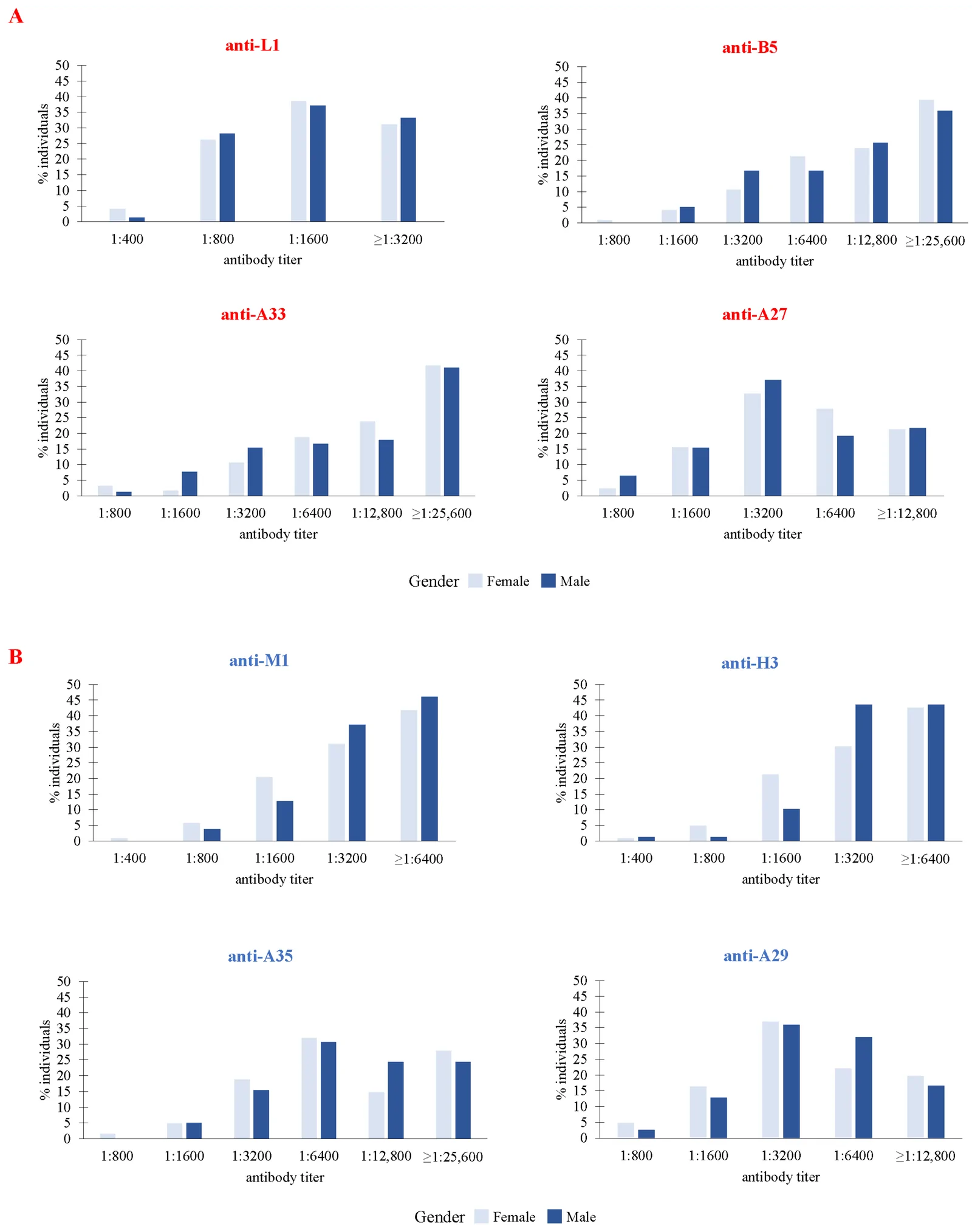
Comparison between the antibody response against VACV and MPXV proteins in females and males in the different age groups. The sera from the individuals of both genders were used to compare the antibody levels against the L1, A33, A27, and B5 VACV proteins (**A**) and the M1, A35, A29, and H3 MPXV proteins (**B**), as well as the population numbers. The antibody responses were similar in women and men against both the VACV and MPXV proteins, with lower titers in most individuals against the L1/M1 proteins. Statistical differences among the groups were not found.

**Table 1:** VACV and MPXV proteins analyzed in this study and references to MPXV mRNA vaccine candidates.

Virus	Investigated Antigen	Virion Form	MPXV mRNA Vaccines			
			mRNA Vaccine	MPXV Antigens	Animal Model	Preclinical or Clinical Phase and References
**VACV**	L1	MV *	mRNA based Mpox	M1, A35	BALB/c mice	Preclinical [37,38]
				B6, A29	BALB/c mice	Preclinical [38]
				M1, B6, A35, A29	BALB/c mice ^unknown mice	Preclinical [31,38,39,40]
				M1, E8, H3, A35, B6, A29	BALB/c mice	Preclinical [32]
	B5	EV **	Rmix 4	M1, B6, A35, A29	BALB/c and C57BL/6 mice	Preclinical [30]
	A33	EV	Rmix 6	M1, H3, E8, B6, A35, A29	BALB/c and C57BL/6 mice	Preclinical [30]
	A27	MV	MPXVac-097	M1, E8, B6, A35, A29	mice	Preclinical [33]
**MPXV**	M1	MV	MPXV-1103	M1, B6, A35, A29	BALB/c mice	Preclinical [41]
	H3	MV	BNT166a	M1, H3, B6, A35	Vaccinia-naïve and vaccinia-experienced volunteers	Clinical I/II [34]
	A35	EV	BNT166c	M1, B6, A35	BALB/c mice cynomolgus macaques	Preclinical [34]
	A29	MV	Moderna mRNA–1769	M1, B6, A35, A29	Vaccinia-naïve volunteers	Clinical I/II [42]

* MV, mature virion; ** EV, enveloped virion.

**Table 2:** Size and demographic distribution of participants *.

Group	Age	Birth Years	Number of Participants
0	20–43	1980–2004	20
A	44–53	1970–1979	50
B	54–63	1960–1969	50
C	64–73	1950–1959	50
D	74–93	1930–1949	50

* The present study was performed in 2023.

## Data Availability

The authors confirm that the data supporting the findings of this study are available within the article.

## Abbreviations

The following abbreviations are used in this manuscript:

DRC	Democratic Republic of Congo
EV	Enveloped virion
MPOX	Monkeypox
MPXV	Monkeypox virus
MV	Mature virion
OPXV	Orthopoxvirus
PHEIC	Public health emergency of international concern
VACV	Vaccinia virus
VARV	Variola virus
WHO	World Health Organization
